# Supplementary material for: Complexome profiling on the Chlamydomonas lpa2 mutant reveals insights into PSII biogenesis and new PSII associated proteins
Source: J Exp Bot. 2021 Aug 26;73(1):245–62. doi: 10.1093/jxb/erab390 (PMC8730698; doi:10.1093/jxb/erab390)
Supplement: erab390_suppl_Supplementary_Dataset_S1 [file erab390_suppl_supplementary_dataset_s1.zip › Supplemental Dataset 1 - Excel List and all profiles/plots/LHCBM8_Cre06.g284250.html]

Trivial name: LHCBM8  
  
Euclidean distance: 5357084.43  
Mean Intensity (WT): 585160.22  
Mean Intensity (Mut): 703460.16  
Distance: 7.62  
  
MapMan: PS.lightreaction.photosystem II.LHC-II  
  
p value of intensity sums Welch test: 0.3946
